# Effect of mucin 4 allele on susceptibility to experimental infection with enterotoxigenic F4 *Escherichia coli* in pigs fed experimental diets

**DOI:** 10.1186/s40104-019-0366-1

**Published:** 2019-07-17

**Authors:** Samantha O. Sterndale, Danica J. Evans, Josephine P. Mansfield, Julie Clarke, Shafi Sahibzada, Sam Abraham, Mark O’Dea, David W. Miller, Jae Cheol Kim, John R. Pluske

**Affiliations:** 1 Agriculture Sciences, College of Science, Health, Engineering and Education Murdoch University, Murdoch, WA 6150 Australia; 2 CSIRO Health and Biosecurity, PO BOX 10041, Adelaide, BC 5000 Australia; 3 Antimicrobial Resistance and Infectious Disease Laboratory, Murdoch University, Murdoch, WA 6150 Australia; 4 AB Vista Asia Pte. Ltd, The Mezzo, Whampoa, 329682 Singapore

**Keywords:** Diarrhoea, *Escherichia coli*, F4, *MUC4*, Weaner pigs, Zinc oxide

## Abstract

**Background:**

This study investigated the validity of the DNA-marker based test to determine susceptibility to ETEC-F4 diarrhoea by comparing the results of two DNA sequencing techniques in weaner pigs following experimental infection with F4 enterotoxigenic *Escherichia coli* (ETEC-F4). The effects of diet and genetic susceptibility were assessed by measuring the incidence of piglet post-weaning diarrhoea (PWD), faecal *E. coli* shedding and the diarrhoea index.

**Results:**

A DNA marker-based test targeting the mucin 4 gene (*MUC4*) that encodes F4 fimbria receptor identified pigs as either fully susceptible (SS), partially or mildly susceptible (SR), and resistant (RR) to developing ETEC-F4 diarrhoea. To further analyse this, DNA sequencing was undertaken, and a significantly higher proportion of C nucleotides was observed for RR and SR at the *Xba*I cleavage site genotypes when compared to SS. However, no significant difference was found between SR and RR genotypes. Therefore, results obtained from Sanger sequencing retrospectively allocated pigs into a resistant genotype (*MUC4–*), in the case of a C nucleotide, and a susceptible genotype (*MUC4+*), in the case of a G nucleotide, at the single nucleotide polymorphism site. A total of 72 weaner pigs (age ~ 21 days), weighing 6.1 ± 1.2 kg (mean ± SEM), were fed 3 different diets: (i) positive control (PC) group supplemented with 3 g/kg zinc oxide (ZnO), (ii) negative control (NC) group (no ZnO or HAMSA), and (iii) a diet containing a 50 g/kg high-amylose maize starch product (HAMSA) esterified with acetate. At days five and six after weaning, all pigs were orally infected with ETEC (serotype O149:F4; toxins LT1, ST1, ST2 and EAST). The percentage of pigs that developed diarrhoea following infection was higher (*P* = 0.05) in *MUC4+* pigs compared to *MUC4–* pigs (50% vs. 26.8%, respectively). Furthermore, pigs fed ZnO had less ETEC-F4 diarrhoea (*P =* 0.009) than pigs fed other diets, however faecal shedding of ETEC was similar (*P* > 0.05) between diets.

**Conclusion:**

These results confirm that *MUC4+* pigs have a higher prevalence of ETEC-F4 diarrhoea following exposure, and that pigs fed ZnO, irrespective of *MUC4* status, have reduced ETEC-F4 diarrhoea. Additionally, sequencing or quantifying the single nucleotide polymorphism distribution at the *Xba*I cleavage site may be more reliable in identifying genotypic susceptibility when compared to traditional methods.

## Background

The F4 enterotoxigenic *Escherichia coli* (ETEC-F4) infection model has been used to determine if a dietary intervention (e.g., nutrient, feed additive) can reduce the severity and the consequences associated with the infection [1–3]. However, not all pigs are susceptible to developing an ETEC-F4 infection as this depends, in part, on the presence and amount of F4 receptors found in the brush border membrane of the small intestine. Piglets identified as susceptible have a sufficient amount of F4 receptors in the small intestine (SI), allowing the ETEC to adhere and develop ETEC-F4 diarrhoea [4, 5]. Susceptibility has been identified as the dominant autosomal allele and mapped at the mucin 4 (*MUC4*) gene on chromosome 13 [6]. Previous studies have determined the presence of F4 receptors using an *in vitro* adhesion assay, which requires the piglet to be sacrificed [7, 8]. Alternatively, in experiments that require pigs to be predisposed to developing ETEC-F4 diarrhoea, polymerase chain reaction-restriction fragment length polymorphism testing that targets the *MUC4* gene has been used to determine which pigs are susceptible [4, 9]. Some researchers have further divided these piglets into three genotypes depending on the ETEC adhesion to the brush-border membrane following slaughter: fully susceptible (SS), partially or mildly susceptible (SR), and fully resistant (RR) [8, 10]. Sequencing DNA has identified a single nucleotide polymorphism (SNP) (DQ848681:g.8227C > G), known as the *Xba*I polymorphism, as a possible marker for susceptibility. At the *Xba*I polymorphism, the C allele is associated with resistance and the G allele [6]. However, limited research has been completed analysing for susceptibility using reads of individual DNA strands within a sample (each strand theoretically representing a single cell), comparing the proportion of G to C alleles and then comparing these results to the gel electrophoresis DNA-based marker test as described by [4].

Pigs with genetically susceptible allelic profiles have greater susceptibility to ETEC-F4 infection, and they also tend to have a slower growth rate, prolonged ETEC-F4 diarrhoea and higher faecal ETEC shedding scores when infected [2]. Therefore, it is important to select and allocate experimental pigs to the dietary treatments based on presence/absence of the *MUC4* gene G/C profile to eliminate this confounding factor in nutrition studies using the ETEC-F4 infection model. Mineral compounds such as zinc oxide (ZnO) and copper sulphate (CuSO_4_) have traditionally been used to mitigate post-weaning diarrhoea (PWD); however, due to environmental concerns and risks associated with microbial resistance, different nutritional strategies must be found [11]. Amending the diet of weaned pigs with various dietary fibre types, including resistant starch (RS), is thought to positively influence GIT functions such as fluid and electrolyte uptake, colonic muscular activity, microbial composition, and (or) through production of short-chain fatty acids [12, 13]. In humans hospitalised with acute infectious gastroenteritis, a reduction in duration of diarrhoea after receiving an oral rehydration solution supplemented with acetylated high-amylose maize starch (HAMSA) was seen [14]. This was likely due to the release of esterified acetate from HAMSA promoting fluid and electrolyte uptake in the colon [15], hence there could be some benefit feeding this product to post-weaned pigs with diarrhoea.

This study comprised three hypotheses. First, we hypothesised that sequence analysis to determine presence of G or C nucleotides at the *Xba*I polymorphic site is a valid method to determine potential ETEC-F4 susceptibility. Having done this, we then hypothesised that pigs with the F4 receptor (*MUC4+* allele) would develop more ETEC-F4 diarrhoea than pigs without the F4 receptor (*MUC4–* allele) when experimentally infected with ETEC-F4. Finally, we reasoned that feeding a diet supplemented with an acetylated high-amylose maize starch (HAMSA) or zinc oxide (ZnO) will decrease the incidence of ETEC-F4 associated diarrhoea in weaned pigs experimentally infected with ETEC-F4.

## Methods

This experiment was approved by the Animal Ethics Committee of Murdoch University (R2812/16).

### Animals, housing, experimental design and diets

This study used samples taken as part of another study [16] that examined the influence of different diets fed after weaning on diarrhoea, blood and production variables. Accordingly, only data pertaining to relationships between susceptibility or resistance of pigs, as assessed by the presence or absence of the *MUC4* mutation, in relation to the diets fed was examined.

At 21 days of age, 72 male castrate pigs (Large White × Landrace) weighing 6.1 ± 1.2 kg (mean ± SEM) were weaned from a commercial piggery in Western Australia. The pigs arrived at Murdoch University in two batches, three days apart, with all pigs subject to the same experimental timeframe. On arrival pigs were randomly allocated into their experimental treatment group in six replicate pens (four pigs per pen) using a randomised block distribution based on live weight at weaning (three treatments × six replicates per treatment × four pigs per pen; *n* = 72). Pigs were housed in three different rooms at a temperature of 28.0 ± 1.0 **°**C in pens of metal construction with plastic floors, allowing at least 0.6 m^2^ per pig. Each room had two pens per treatments, with six pens in total. The pens were fitted with a nipple drinker, five space feeder and plastic bottles for enrichment purposes.

The study was a factorial arrangement of treatments with the factors being (a) the genetic susceptibility to developing ETEC-F4 associated diarrhoea (*MUC4*+/−) and (b) three diets: (i) positive control (PC), which comprised the base diet with 3 g ZnO/kg added (ii) negative control (NC), which consisted of the base diet without ZnO or HAMSA, and (iii) the base diet with 50 g HAMSA/kg replacing 50 g/kg wheat. The base diet was formulated to meet the animals’ requirements according to the National Research Council (NRC, 2012). Diet compositions and analysed gross energy and nutrient contents are presented in Table 1. The diets, along with water, were offered on an ad libitum basis for 3 weeks after weaning.

**Table 1:** Composition of experimental diets (% as fed basis)

Ingredient	Negative control	Positive control	HAMSA^a^
Barley	10	10	10
Wheat	44.6	44	38.5
Soybean meal	15	15	15
Bloodmeal	1.7	1.7	2
Fishmeal	8	8	8.6
Whey powder	15.9	15.9	15.9
Canola oil	3	3.1	3.3
*L*-Lysine	0.29	0.28	0.25
*DL*-Methionine	0.24	0.24	0.24
*L*-Threonine	0.13	0.13	0.12
*L*-Tryptophan	0.07	0.07	0.07
Vitamin/mineral premix^B^	0.15	0.15	0.15
Limestone	0.46	0.46	0.41
Dicalcium phosphate	0.22	0.22	0.18
Salt (NaCl)	0.2	0.2	0.2
Choline chloride (60%)	0.045	0.046	0.5
Zinc oxide	0.0	0.3	0.0
High amylose corn starch^C^	0.0	0.0	50
Calculated composition			
NE, MJ/kg	10.15	10.16	10.14
Protein	21	21	21
Fat	4.84	5.02	5.20
NDF^d^	8.9	8.8	0.16
ADF^e^	2.7	2.7	2.5
Calcium	0.9	0.9	0.9
Digestible phosphorus	0.68	0.68	0.67
Total lysine	1.36	1.36	1.36
SID^f^ lysine	1.35	1.35	1.35
SID Met+ cysteine	0.81	0.81	0.81
SID threonine	0.85	0.85	0.85
SID tryptophan	0.297	0.297	0.297
SID isoleucine	0.77	0.77	0.77
SID leucine	1.44	1.44	1.46
Analysed composition			
Dry matter	92.4	92.1	92.3
Gross energy, MJ/kg	0.017	0.017	0.017
Protein	22.0	20.9	21.3
Crude fibre	2.7	3.9	2.6
Starch	35.3	34.5	35.4
NDF	8.4	9.0	9.6
ADF	3.4	2.6	3.2
Zinc, ppm	122	1955	458

^a^HAMSA: acetylated high-amylose maize starch

^B^Provided the following nutrients (per kg of air-dried diet): vitamins: A, 7000 IU; D_3_, 1400 IU; E, 20 mg; K, 1 mg; thiamine, 1 mg; riboflavin, 3 mg; pyridoxine, 1.5 mg; cyanocobalamin, 15 μg; calcium pantothenate, 10.7 mg; folic acid, 0.2 mg; niacin, 12 mg; biotin, 30 μg. Minerals: Co, 0.2 mg (as cobalt sulfate); Cu, 10 mg (as copper sulfate); iodine, 0.5 mg (as potassium iodine); iron, 60 mg (as ferrous sulfate); Mn, 40 mg (as manganous oxide); Se, 0.3 mg (as sodium selenite); Zn, 100 mg (as zinc oxide); BJ Grower 1, BioJohn Pty Ltd., WA, Australia

^c^High amylose maize starch; product supplied by CSIRO, Adelaide, Australia

^d^*NDF* neutral detergent fibre

^e^*ADF* acid detergent fibre

^f^*SID* standardised ileal digestible

### Feed analysis

Diet samples were analysed for dry matter, gross energy, crude protein, crude fibre, calcium, phosphorous, neutral detergent fibre, acid detergent fibre, starch and zinc as per protocols for InVivo Labs (Vietnam). Dry matter content was determined using method EC 152/2009. The N content was determined using combustion method 2001.11 [17] and crude protein content was calculated as N content × 6.25. The ADF and NDF contents were determined using the ANKOM Technology methods 8 and 9 respectively [18]. Gross energy content was determined using a ballistic bomb calorimeter (SANYO Gallenkamp, Loughborough, UK). Zinc content was determined using atomic absorption spectroscopy (AAS11 152/2009/EEC).

### DNA sample collection

Bristles and the attached follicles were collected from all 72 pigs on the day of weaning. This was done by gently restraining the piglets, sanitizing the area behind the ear using 70% ethanol wipes, pulling 10–20 bristles including the follicle, and placing the sample into sterile tubes on ice. Utensils and gloves were cleaned between each sample to ensure no cross contamination of follicular cells occurred.

### DNA extraction and the marker-based test

The DNA was extracted from the follicles using a DNeasy Blood and Tissue kit (Qiagen, Australia) according to the manufacturer’s instructions. The DNA concentration was measured using a Nanodrop 2000 spectrophotometer (Thermo Fisher Scientific, Massachusetts, USA). To determine absence or presence of the *MUC4* allele, a polymerase chain reaction-restriction fragment length polymorphism (PCR-RFLP) assay was completed on 25 ng of genomic DNA in a total volume of 25 μL, using 5 μL MyTaq Red Reaction buffer, 0.5 units of MyTaq HS DNA polymerase (Bioline, New South Wales, Australia) and 0.4 μmol/L of each *MUC4* primer: 5′-GTCCCTTGGGTGAGAGGTTA/ 5′-CACTCTGCCGTTCTCTTTCC (Sigma-Aldrich, Australia). Thermocycling was performed as described by Jensen et al. [5]. Restriction enzyme digest with *Xba*I (Promega, Wisconsin, USA) to identify polymorphism on 5 μL PCR product was completed overnight at 37 °C and then run on a 2% agarose gel with GelRed (Biotium, California, USA) using 100 bp Gene ruler (Thermo Fisher Scientific, Massachussetts, USA) by electrophoresis for 120 min at 80 V. Bands were visualised on a BioRad GelDoc (Life Science, California, USA) with resistant alleles viewed as a single band at 367 bp and susceptible alleles viewed as two bands at 151 and 216 bp.

### DNA sequence analysis of MUC4

To confirm absence or presence of the *MUC4* mutation, Sanger sequencing was completed on all the DNA samples to further analyse and/or confirm the genotype (*MUC4+/−*) of the pigs. Pigs were classified as resistant (*MUC4–*) if a C nucleotide was present at the *Xba*I polymorphism site and susceptible (*MUC4+*) in case of a G nucleotide [6]. For verification purposes, a total of 60 DNA samples collected were analysed to compare the three different genotypes as determined by the gel electrophoresis DNA-marker test, from the same commercial farm but across different maternal/sire lines (Fully Resistant (RR): *n* = 22, Susceptible Resistant (SR): *n* = 28, Fully Susceptible (SS): *n* = 10). For quantification of genotypes, next generation sequencing (NGS) was undertaken. For preparation of NGS samples, amplification of the *MUC4* allele was undertaken using the primers MUC4-Illumina 5′-TCGTCGGCAGCGTCAGATGTGTATAAGAGACAGGTGCCTTGGGTGAGAGGTTA/− 5′-GTCTCGTGGGCTCGGAGATGTGTATAAGAGACAGCAACCCCATGAAGGAGATC, which contained Illumina adapter sequences on the 5′ ends (italicised) and amplified a 257-bp product spanning the *MUC4* allele. Library preparation was performed using a Nextera XT library kit (Illumina) as per manufacturer’s instructions, and sequencing was performed on a NextSeq 500 using a V2 2 × 150 flowcell. Results were analysed using Geneious (Version 11.1.3). For determination of *MUC4* genotype, sequenced reads were compared to GenBank accession DQ848681 [5]. For quantification, all NGS reads for a given sample were aligned to reference sequence DQ848681, and the presence of C or G nucleotide polymorphisms at the *Xba*I polymorphism site calculated and expressed as percentages.

### ETEC-F4 infection model

On days five and six after weaning pigs were infected with an enterotoxigenic *E. coli* strain (*ETEC*; serotype O149:F4; toxins LT1, ST1, ST2 and EAST). Briefly, an aliquot from stock ETEC-F4 stored at − 80 °C was grown on a Tryptic Soy Agar (TSA) with 5% sheep blood (Thermo Scientific, Australia) overnight at 37 **°**C. A single colony with clear haemolysis was selected and added to 20 mL of sterile Tryptic Soy Broth (TSB) (Bacto Tryptic soy broth; Becton, Dickinson and Company, USA) and incubated in a water bath overnight at 23 **°**C. The culture was centrifuged at 2,000×*g* for 15 min, the supernatant discarded and the pellet resuspended in 20 mL fresh TSB. From this suspension 4 mL was added to 400 mL of TSB and further incubated for 3.5 h at 37 **°**C with orbital shaking at 120 r/min. The culture was centrifuged at 2,000×*g* for 15 min, the supernatant discarded and the pellets resuspended in fresh cold TSB. An aliquot was taken to measure the concentration of viable bacteria. The culture was kept on ice and all piglets were orally dosed with 9 mL of 1.03 × 10^9^ colony forming units (CFU/mL) on days five and six after weaning. Oral gavage was performed by restraining the piglet and administering the inoculum via a drenching gun.

### Faecal consistency score, ETEC-F4 diarrhoea, and faecal β-haemolytic ETEC shedding

Faecal consistency was visually assessed for each piglet daily for the 21 days of the study using a four-point scale, as follows: score (1) firm, (2) soft, spreads slightly, (3) soft and loose, (4) watery liquid consistency. Pigs with a score 4 between days six and fourteen were identified as having ETEC-F4 diarrhoea. On days zero, five, six, seven and nine after weaning, faecal rectal swabs were taken to determine the shedding of β-haemolytic ETEC-F4. These swabs were plated on TSA 5% sheep blood agar plates (Thermo Scientific, Thebarton, Australia), incubated overnight at 37 **°**C and visually assessed and compared to the ETEC-F4 used in the infection, the following day for the presence of β-haemolytic colonies. The plates consisted of 5 sections and given scores from 0 to 5, where 0 has no growth and 5 is the highest possible growth [19]. Total faecal ETEC shedding score was calculated as the sum of all swab plate scores from days zero, five, six, seven, and nine.

## Statistics

All statistical analyses were completed using statistical packages SPSS v. 24 (IBM SPSS, USA) and R [20]. Total *E. coli* faecal shedding scores (swab scores) were analysed using a generalized linear model with pen as a covariate, and analysed the effects of the presence or absence of F4 fimbria receptors (*MUC4+/MUC4–*), the three dietary treatments, and their interaction. The percentage of pigs that developed ETEC-F4 diarrhoea for two consecutive days (score 4) between days six and fourteen was analysed using Chi-square tests. Diarrhoea index (DI) was measured as number of days the pig had score 4 diarrhoea, and expressed as the proportion of days with diarrhoea over nine days after weaning (i.e., between days six and fourteen). The data were not normally distributed and transformation did not correct this. Therefore, raw means are presented and the significance was tested using both the non-parametric Kruskal-Wallis test and Mann-Whitney U test. The relationship between the DI and diet was analysed using Kruskal-Wallis test to statistical differences between treatment groups. Mann-Whitney U test was used to analyse the differences between *MUC4+/−* and the DI. Due to the abnormal distribution of the data and the type of data interactions between F4 fimbria and the dietary treatments for diarrhoea and DI could not be analysed. Significant differences were accepted at *P <* 0.05, and trends were considered when 0.05 *< P <* 0.1.

Analyses on *MUC4 +/−* was completed using generalised logistic regression models (GLM) involving proportional response variables in R [20]. An association between the NGS results that determined the presence of a C or G nucleotide at the *MUC4* site and the gel electrophoresis DNA-marker based test were investigated using the above models. The GLM models were evaluated, and the models were specified with quasibinomial family for error distribution by considering the overdispersion concerns. The overall model fit was assessed using a likelihood ratio test that derives the *P*-values using a χ^2^ distribution. Odds ratios (OR) and confidence intervals (CI) were calculated from the logistic regression models by exponential transformation of the coefficients and its intervals using commands *coef* and *cofint.* Associations were also assessed for C or G nucleotides with developing ETEC-F4 diarrhoea in pigs using separate univariable analyses.

## Results

### MUC4 analysis

**Fig. 1:**
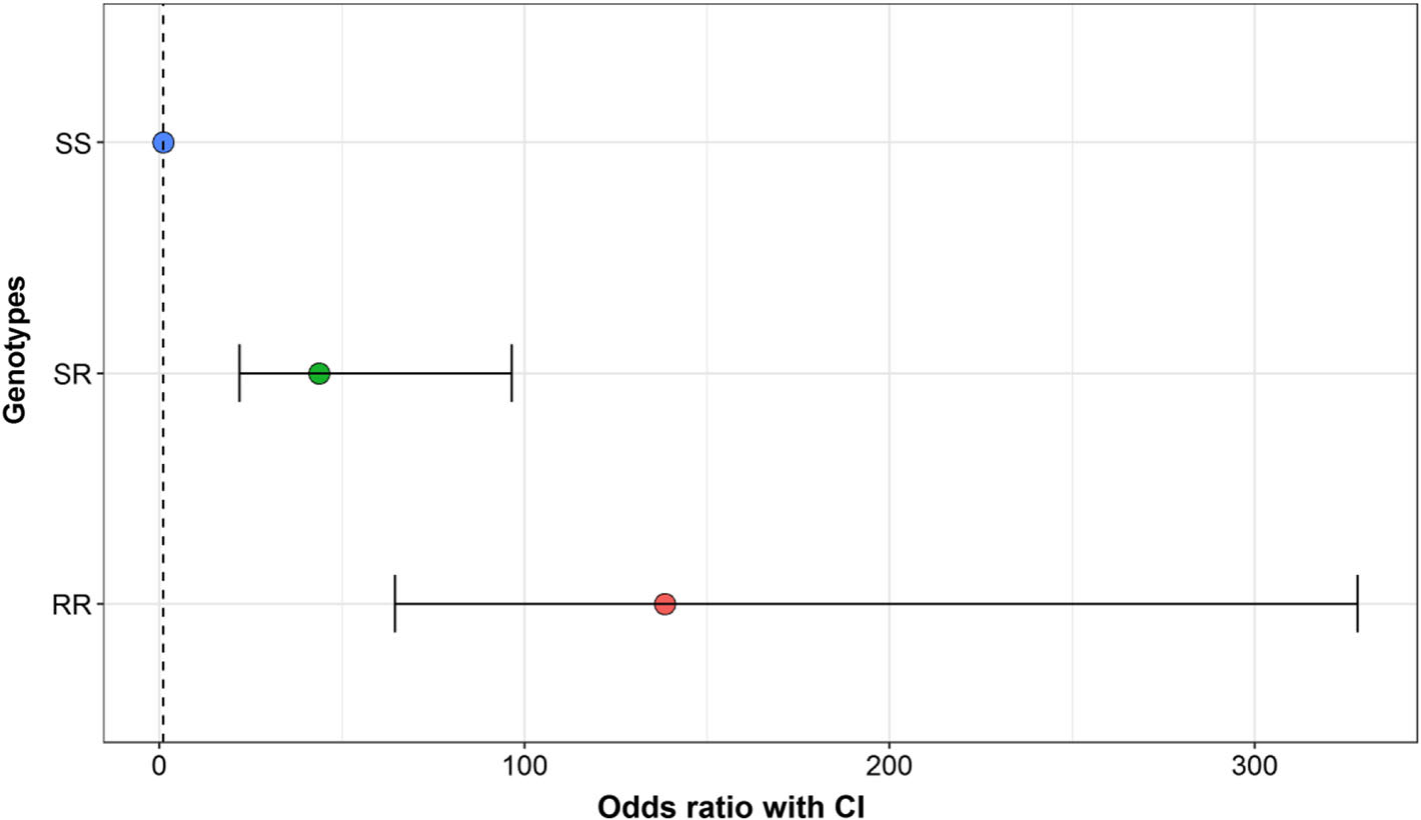
The plot showing the odds ratio and 95% CI of the three genotypes. The dotted vertical line shows an odds-ratio of 1 (no effect). Data were analysed using the univariable model to determine if there was an association between the percentage of C to G nucleotides as determined by NGS and genotype as determined by the PCR-RFLP test

Testing of DNA for the *Xba*I polymophism using the PCR-RFLP method showed that of the 72 pigs tested, 51 were found to be fully susceptible or partially susceptible (SS/SR). The distribution of these pigs was 16 in PC, 18 in NC and 17 pigs in the HAMSA-fed treatments. To further analyse the genotype of each pig, the results obtained by Sanger sequencing and NGS were compared to the PCR-RFLP method. Sanger sequencing had the ability to identify either a C or G neuceoltide at the *MUC4* site, whereas NGS could identify the proportion of C to G nucleotides present at the same site. The results confirmed that pigs with a C nucleotide by Sanger sequencing also had more than 50% C nucleotides via NGS at the *MUC4* site, and were therefore classified as resistant. Using univariable analysis, a significant association (*P* < 0.001) was identified between the percentage of C to G nucleotides as determined by NGS and genotype as determined by the PCR-RFLP test. A higher (*P <* 0.001) proportion of C nucleotides was observed for RR and SR genotypes when compared to SS. However, no difference (*P >* 0.05) was found between SR and RR genotypes (Fig. 1). Therefore, pigs were retrospectively allocated into resistant (*MUC4–*)*,* in the case of a C nucleotide, and susceptible (*MUC4+*), in the case of a G nucleotide, at the *MUC4* gene (Fig. 1). From the 72 pigs tested, a total of 16 pigs were *MUC4+* and distributed into treatments as 5, 4 and 7 for pigs fed diets PC, NC and HAMSA, respectively.

One *MUC4+* piglet fed the PC diet and a *MUC4–* piglet fed the HAMSA diet died during the experiment due to severe non-haemolytic *E. coli* ileitis, and one piglet (*MUC4+* fed the PC diet) was removed due to lameness. The data for these pigs were removed from the dataset.

### ETEC-F4 diarrhoea and faecal β-haemolytic ETEC shedding

**Fig. 2:**
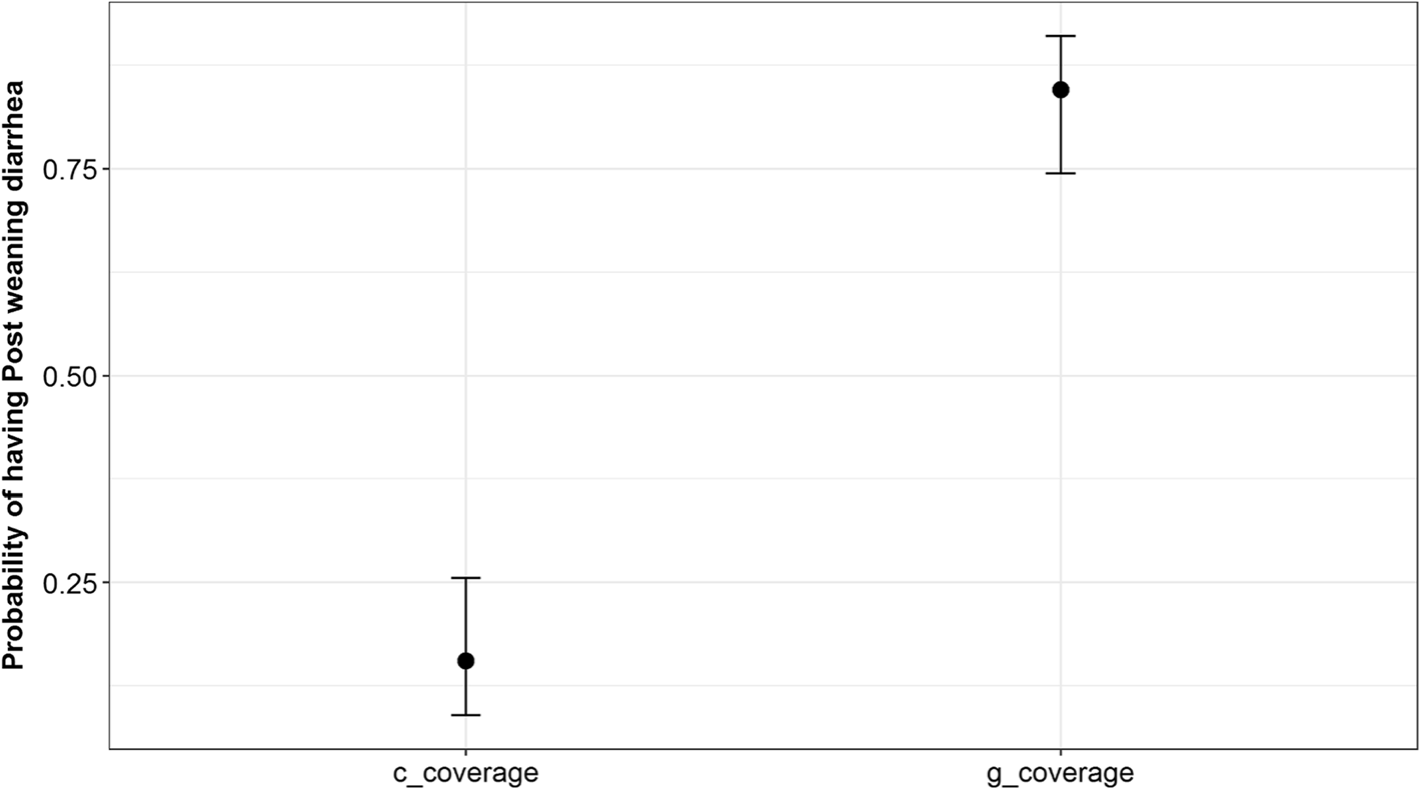
Predicted probability of the percentage of C/G nucleotides present at the *Xba*I polymorphism and the ability to predict ETEC-F4 diarrhoea. Data were analysed using the univariable model

In total, 8.8% of pigs fed the PC diet, 50% of pigs fed the NC diet, and 34.8% of pigs fed the HAMSA diet developed ETEC-F4 diarrhoea. A significant difference in diarrhoea was observed (*P* = 0.048) between the PC and NC diets; however, no difference (*P* > 0.05) was noted between pigs fed diets HAMSA and PC, or pigs fed diets HAMSA and NC. Pigs that were classified as *MUC4+* had a higher percentage of diarrhoea (*P* = 0.05) when compared to *MUC4–* pigs (50% and 26.8%, respectively). Using the univariable model the probability of developing ETEC-F4 diarrhoea was highly associated (*P* < 0.001) with the proportion of C or G nucleotides present at the *Xba*I polymorphism (Fig. 2). The interaction between ETEC-F4 diarrhoea, treatments and genotype could not be analysed due to the abnormal distribution of the data.

Pigs fed diet PC had a lower diarrhoea index (DI) compared to the NC and HAMSA groups (1.97 vs 14.2 and 14.3, *P* = 0.02). Pigs fed HAMSA had a DI no different to the NC group (*P* > 0.05). Overall, genetically resistant pigs had a DI of 6.2 and susceptible pigs had a score of 14.2 (*P* = 0.05) as determined by Sanger sequencing*.* Although not significant due to the low number of susceptible pigs and high standard deviation, susceptible pigs fed PC had a lower DI compared to the NC and HAMSA groups (2.78 vs 19.4 and 20.4, respectively). The interaction between DI, treatments and genotype could not be analysed due to the abnormal distribution of the data.

Dietary treatment did not influence total β-haemolytic ETEC shedding scores, with the average total ETEC shedding score for diets PC, NC and HAMSA being 3.96, 4.05 and 4.42, respectively (*P* = 0.94). Genotype did not influence ETEC shedding (*P* = 0.43), and no significant association was observed (*P* = 0.38) between ETEC shedding and any of the dietary treatment groups and *MUC4+/MUC4–* genotypes.

## Discussion

The hypotheses tested in this study were that pigs with *MUC4+* allele would develop more ETEC-F4 diarrhoea than pigs with the *MUC4–* allele, sequencing of the *MUC4– *allele would provide a more robust prediction of genotype, and feeding ZnO would reduce PWD. Previous studies have shown that pigs can be susceptible (SS), partially susceptible (SR) or resistant (RR) to developing ETEC-F4 diarrhoea depending on the *MUC4* allele [2, 4, 21]. By comparing the gel electrophoresis DNA marker-based test and DNA sequencing results, it was shown that SR and RR pigs are not significantly different as the percentage of C to G nucleotides present at the *Xba*I polymorphism site does not vary. Pigs that were determined to be SS by the DNA marker-based test were, in fact, significantly different to SR/RR pigs. These results suggest that the PCR-RFLP test does not fully identify the three different genotypes, whereas Sanger sequencing can accurately identify the dominant nucleotide present at the *MUC4* site and therefore genetically susceptible/resistant pigs. Furthermore, using the results from NGS DNA sequencing, the percentage of C or G nucleotides present at the *Xba*I polymorphism site can predict the probability of the pigs developing ETEC-F4 diarrhoea.

Rasschaert et al. [22]compared results completed using an *in vitro* villous assay that could distinguish between the three genotypes to the DNA marker-based test that was used in the current experiment. These authors found that although the DNA marker-based test for *MUC4+* is useful, 30.2% of pigs that tested *MUC4–* were positive in the *in vitro* villous assay for homozygote and heterozygote susceptible (SR/SS). These results suggest that an additional gene (or genes) is (are) likely responsible for the disparity in receptors for F4 *E. coli* [22]*,* and thus ETEC colonisation and disease. In this regard, other authors [23, 24] have demonstrated that the *MUC13* gene can identify the presence of intestinal receptors that predispose pigs to developing *E. coli* F4 infection. In the present study, the percentage of pigs that developed ETEC-F4 diarrhoea, as anticipated, was significantly different between susceptible and resistant pigs, supporting work by Jensen et al. [4] showing that the diarrhoea of genetically susceptible pigs was higher than that of resistant pigs. The same study demonstrated that 87% of susceptible pigs (determined by PCR-RFLP) had ETEC-F4 diarrhoea [4], unlike in the present study where 8 out of the 16 susceptible pigs (determined by Sanger sequencing) had ETEC-F4 diarrhoea. The remaining 50% of susceptible pigs that did not develop diarrhoea could be due to factors including the amount of F4 receptors in the epithelium, delivery method and variation in the dose of ETEC-F4 administered, pH of the stomach, or other luminal factors associated with proliferation of pathogens such as commensal microbiota and competitive elimination [1]. Another cause of variation in the infection response in the current study likely lies with the quantity of intestinal brush-border F4 receptors [22, 25]. Fewer intestinal brush border receptors limit the probability of ETEC-F4 adhering to the small intestine, and therefore reduces ETEC-F4 associated diarrhoea.

Genetically susceptible pigs (*MUC4+*) are expected to replicate more F4 *E. coli* in the small intestine compared to *MUC4–* pigs, leading to greater *E. coli* shedding [26]. As a consequence, these pigs are more susceptible to infection due to the higher colonisation [26] and faecal-oral recycling compared to their negative counterparts. In the current study, total faecal ETEC shedding was not significantly higher in susceptible pigs compared to resistant pigs. Casini et al. [21] showed that on days four and five after ETEC-F4 infection, susceptible pigs had significantly higher faecal shedding and more diarrhoea compared to resistant pigs. This is further supported by another study, where average faecal scores, days with diarrhoea and total *E. coli* shedding were higher (*P <* 0.001) in *MUC4+* pigs compared to *MUC4–* pigs [27]. In the present study there were no significant differences amongst the dietary treatment groups with faecal ETEC shedding. This result did not correlate with the DI or the percentage of pigs with diarrhoea. This demonstrates that although pigs shed ETEC, they may not necessarily present with overt diarrhoea.

The lack of response in faecal ETEC shedding after supplementation of high levels of ZnO in the diet, which contrasts to reduced ETEC-F4 diarrhoea, has also been reported by others [28–32]. In contrast, Slade et al. [33] showed that dietary supplementation of 3,100 ppm ZnO reduced faecal ETEC shedding in challenged pigs. Nevertheless, these findings suggest that the suppression of diarrhoea seen with high levels of ZnO supplementation may not necessarily be associated only with the bactericidal effect of ZnO *per se* [32], but with previous exposure to the bacteria [34], reduced bacterial adherence, anti-inflammation effects, and (or) improved barrier function [35]. Furthermore and due to the low number of *MUC4+* pigs the suppression of ETEC-F4 diarrhoea in pigs fed ZnO could also be due to the low number of genetically susceptible pigs. The lack of an ameliorating effect on PWD of feeding HAMSA could be due to the level of supplementation of HAMSA being inadequate to provide clinical benefit.

## Conclusion

This experiment confirmed that ETEC-F4 diarrhoea was significantly higher in *MUC4+* pigs, as determined by sequence analysis of the gene compared to *MUC4–* pigs following experimental ETEC infection. Furthermore, pigs fed ZnO had less diarrhoea than pigs fed other diets, but this wasn’t reflected in any change in faecal *E. coli* shedding between the diets. In addition, sequencing or quantifying the single nucleotide polymorphism distribution at the *Xba*I cleavage site may be more reliable in identifying genotypic susceptibility when compared to traditional methods. However, further research is needed to increase the infection rate and ETEC shedding in *MUC4+* pigs by determining other factors involved and/or altering the delivery method of the ETEC-F4 to ensure accurate dosage. Furthermore understanding the genetics of ETEC suscpetiblity is also of importance commercially to reduce the impact of disease in production.

## Data Availability

The authors may provide the dataset used and analysed on request.

## Abbreviations

DI: Diarrhoea index
ETEC-F4: F4 enterotoxigenic *Escherichia coli*
GIT: Gastrointestinal tract
HAMSA: Acetylated high-amylose maize starch
MUC4: Mucin 4 gene
MUC4-: Resistant genotype
MUC4 +: Susceptible genotype
NC: Negative control
PC: Positive control
PWD: Post-weaning diarrhoea
RR: Fully resistant
SI: Small intestine
SR: Partially susceptible
SS: Fully susceptible
Zn: Zinc Oxide
