# Correction to: Effect of mucin 4 allele on susceptibility to experimental infection with enterotoxigenic F4 *Escherichia coli* in pigs fed experimental diets

**DOI:** 10.1186/s40104-019-0383-0

**Published:** 2019-08-12

**Authors:** Samantha O. Sterndale, Danica J. Evans, Josephine P. Mansfield, Julie Clarke, Shafi Sahibzada, Sam Abraham, Mark O’Dea, David W. Miller, Jae Cheol Kim, John R. Pluske

**Affiliations:** 1 Agriculture Sciences, College of Science, Health, Engineering and Education, Murdoch University, Murdoch, WA 6150 Australia; 2 CSIRO Health and Biosecurity, PO BOX 10041, Adelaide, BC 5000 Australia; 3 Antimicrobial Resistance and Infectious Disease Laboratory, Murdoch University, Murdoch, WA 6150 Australia; 4 AB Vista Asia Pte. Ltd, The Mezzo, Whampoa, 329682 Singapore

**Correction to: J Anim Sci Biotechnol (2019) 10:56**

**https://doi.org/10.1186/s40104-019-0366-1**

In the original publication of this article [1], the corresponding author wants to change “At the XbaI polymorphism, the C allele is associated with resistance and the G allele [6].” It should be read as “At the XbaI polymorphism, the C allele is associated with resistance and the G allele to susceptibility [6].”
